# Supplementary material for: Nuclei determine the spatial origin of mitotic waves
Source: eLife. 2020 May 26;9:e52868. doi: 10.7554/eLife.52868 (PMC7314552; doi:10.7554/eLife.52868)
Supplement: Transparent reporting form [file elife-52868-transrepform.pdf]

Westbrook Centre, Milton Road
Cambridge CB4 1YG
UK
P 01223 855340
W elifesciences.org
T @elife

eLife's transparent reporting form

We encourage authors to provide detailed information *within their submission* to facilitate the interpretation and replication of experiments. Authors can upload supporting documentation to indicate the use of appropriate reporting guidelines for health-related research (see EQUATOR Network), life science research (see the BioSharing Information Resource), or the ARRIVE guidelines for reporting work involving animal research. Where applicable, authors should refer to any relevant reporting standards documents in this form.

If you have any questions, please consult our Journal Policies and/or contact us: editorial@elifesciences.org.

Sample-size estimation

- You should state whether an appropriate sample size was computed when the study was being designed
- You should state the statistical method of sample size computation and any required assumptions
- If no explicit power analysis was used, you should describe how you decided what sample (replicate) size (number) to use

Please outline where this information can be found within the submission (e.g., sections or figure legends), or explain why this information doesn't apply to your submission:

No explicit power analysis was used. Various cell-free extract experiments were carried out and analyzed afterwards (which did not require sacrificing any animals), which showed clearly that wider tubes have a higher fraction of boundary-driven waves than thinner tubes (Figure 5). For statistical significance, see statistical reporting.

Replicates

- You should report how often each experiment was performed
- You should include a definition of biological versus technical replication
- The data obtained should be provided and sufficient information should be provided to indicate the number of independent biological and/or technical replicates
- If you encountered any outliers, you should describe how these were handled
- Criteria for exclusion/inclusion of data should be clearly stated
- High-throughput sequence data should be uploaded before submission, with a private link for reviewers provided (these are available from both GEO and ArrayExpress)

Please outline where this information can be found within the submission (e.g., sections or figure legends), or explain why this information doesn't apply to your submission:

A table with an overview of all analyzed experiments can be found on the GitHub page which also contains the source code for the simulations. Figure 5 includes experiments with 66 different tubes. 49 of these tubes have a higher concentration of sperm chromatin (pooling tubes from 23 different cell-free extracts), while 17 use the lower concentration of sperm chromatin (pooling tubes from 7 different cell-free extracts). In total, 120 tubes were imaged using 30 different cell-free extracts, but only 66 tube experiments were used in the analyses as the other tubes did either not show any waves and/or did not show sufficient cell cycles.

eLife Sciences Publications, Ltd is a limited liability non-profit non-stock corporation incorporated in the State of Delaware, USA, with company number 5030732, and is registered in the UK with company number FC030576 and branch number BR015634 at the address Westbrook Centre, Milton Road Cambridge CB4 1YG, UK | March 2019

Statistical reporting

- Statistical analysis methods should be described and justified
- Raw data should be presented in figures whenever informative to do so (typically when N per group is less than 10)
- For each experiment, you should identify the statistical tests used, exact values of N, definitions of center, methods of multiple test correction, and dispersion and precision measures (e.g., mean, median, SD, SEM, confidence intervals; and, for the major substantive results, a measure of effect size (e.g., Pearson's r, Cohen's d)
- Report exact p-values wherever possible alongside the summary statistics and 95% confidence intervals. These should be reported for all key questions and not only when the p-value is less than 0.05.

Please outline where this information can be found within the submission (e.g., sections or figure legends), or explain why this information doesn't apply to your submission:

Figures 5A,B show that wider tubes have a higher fraction of boundary-driven waves in both low dilution (A) and high dilution (B). This dependence is checked statistically using Pearson's chi squared test, giving $p<0.02$ in both cases (more precisely, $p=0.0016$ for the data in A and $p=0.0199$ in B).

(For large datasets, or papers with a very large number of statistical tests, you may upload a single table file with tests, Ns, etc., with reference to sections in the manuscript.)

Group allocation

- Indicate how samples were allocated into experimental groups (in the case of clinical studies, please specify allocation to treatment method); if randomization was used, please also state if restricted randomization was applied
- Indicate if masking was used during group allocation, data collection and/or data analysis

Please outline where this information can be found within the submission (e.g., sections or figure legends), or explain why this information doesn't apply to your submission:

Not relevant, no clinical studies and no animal experiments were carried out.

Additional data files ("source data")

- We encourage you to upload relevant additional data files, such as numerical data that are represented as a graph in a figure, or as a summary table
- Where provided, these should be in the most useful format, and they can be uploaded as "Source data" files linked to a main figure or table
- Include model definition files including the full list of parameters used
- Include code used for data analysis (e.g., R, MatLab)
- Avoid stating that data files are "available upon request"

Please indicate the figures or tables for which source data files have been provided:

Source code data is available via github: https://github.com/felixnolet/eLife_paper/
As the actual source data of the videos of the various experiments is very large (Gbs of data), it is not directly provided, but available upon request.
